# Prospective comparison of early interim ^18^F-FDG-PET with ^18^F-FLT-PET for predicting treatment response and survival in metastatic breast cancer

**DOI:** 10.1186/s12885-021-08649-z

**Published:** 2021-08-10

**Authors:** Tzu-Pei Su, Jen-Seng Huang, Pei-Hung Chang, Kar-Wai Lui, Jason Chia-Hsun Hsieh, Shu-Hang Ng, Sheng-Chieh Chan

**Affiliations:** 1 Department of Nuclear Medicine, Keelung Chang Gung Memorial Hospital, 222, Maijin Road, Keelung, 204 Taiwan; 2 Division of Hematology/Oncology, Department of Internal Medicine, Keelung Chang Gung Memorial Hospital, 222, Maijin Road, Keelung, 204 Taiwan; 3 Department of Diagnostic Radiology, Linkou Chang Gung Memorial Hospital and Chang Gung University College of Medicine, 5 Fu-Shin Street, Kueishan, Taoyuan, 333 Taiwan; 4 Division of Hematology/Oncology, Department of Internal Medicine, Linkou Chang Gung Memorial Hospital and Chang Gung University College of Medicine, 5 Fu-Shin Street, Kueishan, Taoyuan, 333 Taiwan; 5 Department of Nuclear Medicine, Hualien Tzu Chi Hospital, Buddhist Tzu Chi Medical Foundation, 707, Section 3, Chung-Yang Road, Hualien, 970 Taiwan; 6 School of Medicine, Tzu Chi University, 701 Section 3, Zhong-Yang Road, Hualien, 970 Taiwan

**Keywords:** Breast cancer, Positron-emission tomography, ^18^F-FDG, ^18^F-FLT, Prognosis

## Abstract

**Background:**

To compare the value of interim ^18^F-FLT-PET and ^18^F-FDG-PET for predicting treatment outcomes in patients with metastatic breast cancer after salvage therapy.

**Methods:**

Patients with metastatic breast cancer received PET/CT using ^18^F-FLT and ^18^F-FDG at baseline, after the 1st and 2nd cycle of systemic chemotherapy. The clinical response was classified according to Response Evaluation Criteria in Solid Tumors 1.1 based on contrast-enhanced CT after 3 months of systemic chemotherapy. The metabolic response on PET was assessed according to European Organization for Research and Treatment of Cancer criteria or PET Response Criteria in Solid Tumors (PERCIST) and was correlated to the clinical response, overall survival (OS), and progression-free survival (PFS).

**Results:**

Twenty-five patients entered final analysis. On ^18^F-FDG-PET, clinical responders after 2 chemotherapy cycles (post-2c) had a significantly greater reduction of maximal standardized uptake value (SUV) and the peak SUV corrected for lean body mass (SULpeak) of the tumor than non-responders (*P* = 0.030 and 0.003). Metabolic response determined by PERCIST on post-2c ^18^F-FDG-PET showed a high area under the receiver operating characteristics curve of 0.801 in predicting clinical response (*P* = 0.011). Patients who were metabolic responders by PERCIST on post-2c ^18^F-FDG-PET had a significantly longer PFS (53.8% vs. 16.7%, *P* = 0.014) and OS (100% vs. 47.6%, *P* = 0.046) than non-responders. Survival differences between responders and non-responders in the interim ^18^F-FLT-PET were not significant.

**Conclusions:**

^18^F-FLT-PET failed to show an advantage over ^18^F-FDG-PET in predicting the treatment response and survival in patients with metastatic breast cancer. Assessment of treatment outcome by interim ^18^F-FDG-PET may aid treatment.

**Trial registration:**

The study was retrospectively registered on 02/06/2020 on Clinicaltrials.gov (identifier NCT04411966).

## Background

Over the past decade, although the new therapeutic agents have become available for patients with metastatic breast cancer (mBC), the median survival of these patients remains dismal, ranging from 10 months to 5 years [1]. Thus, a reliable imaging modality capable of early identification of patients unresponsive to therapy is critical to guide individualized treatment.

The change of tumor size on computed tomography (CT) is the current standard for monitoring tumor response in mBC. However, there are substantial limitations to using an anatomic assessment alone because alterations in tumor size manifest later than those of tumor function. In addition, clinical trials have shown that the response based on size criteria may not be a suitable surrogate to predict survival in breast cancer [2, 3]. In this context, molecular imaging techniques such as positron emission tomography (PET) imaging have been advocated for therapy response evaluation.

^18^F-fluorodeoxyglucose (^18^F-FDG) is used to assess tumor glycolytic metabolism, and is the most commonly used PET tracer. Previous studies have shown that the changes in ^18^F-FDG uptake after one or two cycles of neoadjuvant chemotherapy enables early prediction of the histopathologic response in locoregionally advanced breast cancer patients [4, 5]. However, the performance of ^18^F-FDG-PET is compromised due to false-positive findings caused by infectious diseases in immunocompromised patients or post-therapeutic inflammation around the tumor [6].

^18^F-fluorothymidine (^18^F-FLT) uptake is closely associated with tumor cell proliferation [7]. Previous studies have demonstrated that ^18^F-FLT uptake is associated with treatment response in breast cancer patients [8–11]. Contractor et al. reported that a decrease of ^18^F-FLT uptake early after initiating chemotherapy was predictive of tumor response mid therapy [9]. Because ^18^F-FLT-PET is less affected by lower false-positive rates caused by the inflammatory process in the cancer tissue than ^18^F-FDG-PET [12], it has been considered a more suitable imaging tool in the early assessment of treatment response than ^18^F-FDG-PET. However, the results of comparative studies of ^18^F-FDG-PET and ^18^F-FLT-PET in evaluating therapeutic outcomes are inconsistent across different cancers [13–15]. In mBC, the superiority of ^18^F-FLT-PET over ^18^F-FDG-PET in the early prediction of chemotherapy response remains undetermined.

Therefore, we conducted this prospective study to compare the performance of interim ^18^F-FLT-PET and ^18^F-FDG-PET in predicting treatment response and survival outcome in patients with mBC undergoing systemic therapy.

## Methods

### Patients

This was a prospective, single-center trial that compared the performance of interim ^18^F-FLT-PET and ^18^F-FDG-PET in patients with mBC undergoing systemic therapy. The eligible criteria for the patients’ inclusion were as follows: 1) histological diagnosis of breast carcinoma; 2) presence of metastatic cancer—either primary or recurrent cases—based on the pathological or imaging findings. Patients were excluded if they were pregnant, lactating, had simultaneous second primary cancer, or uncontrolled intercurrent illness that would limit their compliance with the study.

The disease staging and treatment protocols of all enrolled patients were reviewed and confirmed by the breast cancer committee at our institute. The committee members included two breast surgeons, three medical oncologists, two radiation oncologists, one radiologist, two nuclear medicine physician and one pathologist. We adhered to standard treatment protocols in accordance with institutional guidelines. Chemotherapy regimens including anthracycline plus docetaxel, paclitaxel plus gemcitabine, anthracycline plus cyclophosphamide, or vinorelbine plus platinum, were contingent upon the decision made by the treating physician. The patients also received additional hormone therapy or trastuzumab-based therapy according to their estrogen receptor (ER), progesterone receptor (PR), or HER-2 status, respectively. Hormone therapy was not administered if patients experienced endocrine therapy failure or if they were diagnosed with potential visceral crisis.

From January 2014 to August 2017, 32 patients were enrolled in this study (clinicaltrials.gov identifier NCT04411966). The study participants underwent whole-body PET/CT imaging (^18^F-FDG and ^18^F-FLT) at baseline. Baseline CT was performed a median of 9 days (range 3–74) before the initiation of systemic therapy. Baseline PET was performed a median of 4 days (range 3–21) before the initiation of therapy. The patients received chemotherapy (68% received docetaxel-based treatment). Nine and five patients received additional hormone therapy or trastuzumab-based therapy according to their estrogen receptor (ER), progesterone receptor (PR), or HER-2 status, respectively. Four patients with bone and abdominal wall metastases received additional radiotherapy for these local lesions. Hormone therapy was dismissed in six patients with a positive ER status due to potential visceral crisis and in three patients with a history of endocrine therapy failure [16].

Interim ^18^F-FDG-PET and ^18^F-FLT-PET were performed a median of 21 days (range 10–27) after the first cycle of chemotherapy (before the start of the second cycle of chemotherapy) and a median of 20 days (range 10–48) after the second cycle of chemotherapy (before the start of third cycle of chemotherapy). ^18^F-FDG-PET and ^18^F-FLT-PET were performed on separate days. The minimum time between both the scans was > 20 h. The clinician was not blinded to the PET results, but the treatment was not allowed to be changed after ^18^F-FLT-PET or interim PET. Contrast-enhanced CT (CE-CT) was performed both at baseline and 3 months after the start of systemic therapy.

### ^18^F-FLT-PET and ^18^F-FDG-PET

Prior to ^18^F-FDG or ^18^F-FLT injection, patients were instructed to fast for at least 6 h. Blood glucose levels were < 150 mg/dL in all participants. The injected dose for each patient scan was 185 ± 10% MBq of ^18^F-FLT and 370 ± 10% MBq of ^18^F-FDG. Each patient underwent both ^18^F-FLT and 18F-FDG PET on two separate days. The PET imaging was performed using a PET/CT system (Discovery STE, GE Health Systems, Milwaukee, WI, USA). Before PET acquisition, helical CT was performed from the head to the proximal thigh according to a standardized protocol with the following settings: transverse 3.75 mm, collimation 1.25 × 16 modes, 120 kVp, smart mA (25–300 mA), 0.5 s tube rotation, 27.5 mm/s table speed, and pitch 1.375. We did not administer intravenous iodinated contrast agents. CT data were resized from a 512 × 512 matrix to a 128 × 128 matrix to match the PET data in order to fuse images and generate CT-based transmission maps. Subsequently, we acquired emission scans from the head to the proximal thigh 50–70 min after injection of ^18^F-FDG or ^18^F-FLT using the three-dimensional mode with 2.5 min per table position. The PET images were reconstructed using the CT data for attenuation correction with an ordered-subset expectation maximization iterative reconstruction algorithm (2 iterations and 28 subsets).

### PET imaging analysis and assessment of metabolic response

We evaluated the PET images in transaxial, sagittal, and coronal planes using a dedicated workstation. The standardized uptake value (SUV) for the metastatic tumor was calculated using the following formula: (measured activity concentration [Bq/mL]) / (injected activity [Bq] / body weight [kg] × 1000). We measured the maximum SUV (SUVmax) and the peak SUV corrected for lean body mass (SULpeak) within a region of interest (ROI) [17]. During the course of chemotherapy, changes in SUV_max and SULpeak_ in target lesions were calculated by comparing radiotracer uptake at time points t_0_, t_1_ and t_2_, as: ΔSUVmax (t_i_) = 100 x [SUVmax (t_i_) – SUVmax(t_0_)] / SUVmax(t_0_), ΔSULpeak (t_i_) = 100 x [SULpeak (t_i_) – SULpeak(t_0_)] / SULpeak (t_0_), where i = cycle of chemotherapy.

To facilitate a direct comparison, we used the European Organization for Research and Treatment of Cancer (EORTC) criteria and PET Response Criteria in Solid Tumors (PERCIST) to evaluate the metabolic response in both ^18^F-FDG-PET and ^18^F-FLT-PET. The metabolic response by the EORTC criteria was based on the same ROI volumes sampled on baseline and interim scans [18]. We defined partial metabolic response (PMR) after one and two chemotherapy cycles as a decrease of ≥15% and ≥ 25% SUVmax, respectively. Stable metabolic disease (SMD) was determined if there was either an increase or decrease of < 15% or < 25% SUVmax after one or two chemotherapy cycles, respectively. Finally, progressive metabolic disease (PMD) was diagnosed as an increase in SUVmax of > 25%.

In accordance with the PERCIST criteria [19], we measured the mean SUL and standard deviation of the SUL in a 3-cm diameter spherical volume of interest (VOI) in the right hepatic lobe for background activity. We evaluated the change in the SULpeak between the most obvious single tumor lesion at the baseline and interim imaging studies in order to determine if the target lesions were different between the two studies. Complete metabolic response (CMR) was determined if complete abrogation of tumor FDG-uptake was observed; PMR was defined as a reduction in SULpeak greater than 30%. PMD was diagnosed as either an increase in SULpeak of at least 30% or the development of a new lesion. SMD was determined if CMR, PMR, and PMD were not present.

The tumor to background ratio was obtained from baseline images. A rectangular region of interest was positioned around the tumor activity in the coronal images with maximum tumor activity. An identical region of interest was placed around comparable unaffected tissue on the contralateral side representing background activity. In the patient with a lumbar spine tumor, a comparable unaffected spine segment was used. We measured the SUVs and SULs of the metastatic lesion and the background tissue to obtain the ratio.

### Clinical response

Clinical response was assessed through an independent assessment of contrast-enhanced CT images obtained three months after the start of chemotherapy compared to the baseline scans. All contrast-enhanced CT images were interpreted blind to the results of the PET. We diagnosed the tumor response on CT as progressive disease (PD), complete response (CR), stable disease (SD), or partial response (PR) according to the Response Evaluation Criteria in Solid Tumors (RECIST), version 1.1 [20]. We classified patients with CR or PR as clinical responders, and those with SD or PD as clinical non-responders.

### Statistical analysis

The Mann–Whiney test was used to compare the ΔSUVmax (t_i_) and ΔSULpeak (t_i_) in the different response groups. The clinical response was defined as the reference standard for PET metabolic response. The predictive power of the metabolic response was assessed by estimating the area under the ROC curve (AUC). The 95% confidence intervals (CI) for AUC and the *P* value of the test of the null hypothesis that AUC = 0.5 (no predictivity) were estimated using bootstrap methods with 1000 replications. Delong’s method was used to test if the observed AUC was significantly greater than 0.5 with the one-sided *P* value [21]. Progression-free survival (PFS) was calculated from the date of inclusion in the study to disease recurrence or progression. Overall survival (OS) was calculated from the date of inclusion in the study to the date of death from any cause or last follow-up. Associations between metabolic response or SUVmax and survival outcome were described graphically using Kaplan–Meier product limit curves and assessed by the log-rank test. All calculations were performed using the SPSS version 21 statistical package (SPSS Inc., Chicago, IL, USA) and MedCalc version 19.1.5 (MedCalc Software, Ostend, Belgium). Two-tailed *P* values < 0.05 were considered statistically significant.

## Results

### Baseline patient characteristics and clinical treatment response

A total of thirty-two patients were enrolled in this study. Seven patients were excluded from the final analysis: five died before completion of chemotherapy and did not complete the interim PET studies. Two were lost to follow-up. The data of the 25 patients that were included in the final analysis are presented in Table 1. The median age of the study participants was 52 years (range, 27–67 years). The metastatic lesions were histopathologically proven in 13 patients, while the lesions in another 12 patients were determined to be metastatic because of the disseminated findings in the images. The median follow-up time for the whole cohort was 38 months (range, 6–61 months). At the end of the follow-up period, 11 patients had died. One patient (4%) had CR, 11 (44%) PR, 6 (24%) SD, and 7 (28%) PD. In patients with PD, five (patient no. 5, 7, 13, 20, and 22) had newly-developed metastatic tumors in the liver, chest wall, and axillary lymph nodes, and the other two (patient no. 6 and 14) had pre-existing metastatic lesions that had enlarged in size.

**Table 1:** Patient characteristics

Patient	Age (yrs)	Primary or Recurrent	Metastatic site	Histology^a^	ER/PR/HER-2^a^	Ki-67^a^	Grade^a^
1	59	Recurrent	Bone, lymph node, other	Ductal	+/−/+	NA	III
2	47	Recurrent	Bone, lymph node	Ductal	+/+/−	10	I
3	40	Recurrent	Lung, liver, lymph node	Ductal	+/+/−	5	II
4	27	Primary	Lung, lymph node	Ductal	+/+/−	10	II
5	52	Recurrent	Bone, lymph node	Ductal	+/−/−	NA	NA
6	50	Recurrent	Bone, liver	Ductal	+/−/+	NA	II
7	54	Recurrent	Lung, lymph node	Ductal	−/−/−	NA	NA
8	62	Recurrent	Bone, other	Ductal	−/−/−	30	III
9	37	Recurrent	Bone	Ductal	+/+/−	40	II
10	48	Recurrent	Bone	Ductal	+/+/−	NA	II
11	67	Primary	Bone	NST	+/+/−	10	I
12	43	Recurrent	Bone	Ductal	−/−/−	NA	III
13	55	Recurrent	Lymph node	NST	+/+/+	NA	NA
14	38	Recurrent	Bone, lung, liver	Ductal	+/+/−	NA	N A
15	61	Primary	Bone, lung	NST	+/+/−	30	II
16	59	Recurrent	Bone	Ductal	+/−/−	10	III
17	52	Recurrent	Bone	Ductal	+/+/+	NA	NA
18	37	Recurrent	Bone, lung, liver, other	NST	+/+/+	50	III
19	50	Recurrent	Bone	Ductal	+/−/−	NA	NA
20	59	Recurrent	Other	Ductal	−/−/−	30	III
21	55	Recurrent	Bone, lung, liver	Ductal	−/−/−	20	II
22	57	Recurrent	Lymph node, other	NST	−/−/−	40	III
23	45	Recurrent	Other	NST	+/+/−	15	NA
24	51	Recurrent	Lung, liver	Ductal	+/+/+	20	II
25	54	Recurrent	Other	NST	−/−/−	> 90	III

^a^ Acquired at diagnosis

Abbreviations: *ER* estrogen receptor, *PR* progesterone receptor, *HER-2* human epidermal growth factor receptor 2, *NA* not available, *NST* no special type

### Semiquantitative measurements on ^18^F-FLT-PET and ^18^F-FDG-PET and their relationships to clinical response

The median SUVmax (± interquartile range) of the target tumors on baseline ^18^F-FLT-PET and ^18^F-FDG-PET was 11.6 (± 11.9) and 11.2 (± 20.5), while the median SULpeak (± interquartile range) of the target tumors on baseline ^18^F-FLT-PET and ^18^F-FDG-PET was 8.83 (± 14.5) and 7.80 (± 12.0). The median ΔSUVmax(t_1_) on ^18^F-FLT-PET and ^18^F-FDG-PET was − 11.8% (median absolute decrease of − 0.2) and − 17.5% (median absolute decrease of − 2.4), while the median ΔSULpeak(t_1_) on ^18^F-FLT-PET and ^18^F-FDG-PET was − 4.4% (median absolute decrease of − 0.1) and − 17.1% (median absolute decrease of − 1.3). The median ΔSUVmax(t_2_) on ^18^F-FLT-PET and ^18^F-FDG-PET was − 26.8% (median absolute decrease of − 0.2) and − 28.5% (median absolute decrease of − 4.1), while the median ΔSULpeak(t_2_) on ^18^F-FLT-PET and ^18^F-FDG-PET was − 6.3% (median absolute decrease of − 0.2) and − 31.9% (median absolute decrease of − 2.4), respectively.

**Fig. 1:**
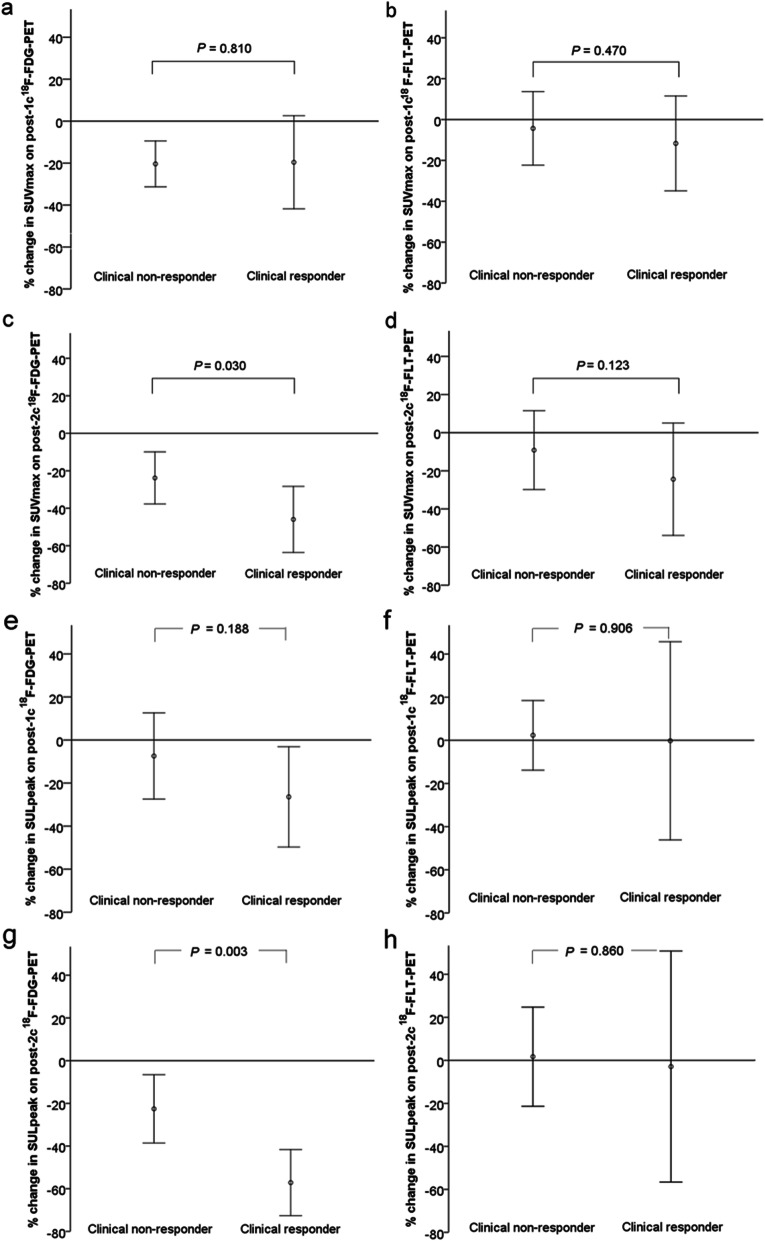
Comparison of SUV and SUL changes between clinical responders and non-responders in patients with metastatic carcinoma. Clinical responders showed significant larger SUV and SUL changes on post-2c ^18^F-FDG-PET (*P* = 0.030 and 0.003) than non-responders

The SUVmax values on baseline ^18^F-FDG-PET and ^18^F-FLT-PET did not differ significantly between clinical responders and non-responders (*P* = 0.744 and 0.785, respectively). Meanwhile, the SULpeak values on baseline ^18^F-FDG-PET and ^18^F-FLT-PET did not differ significantly between clinical responders and non-responders (*P* = 0.830 and 0.378, respectively). Figure 1 depicts the SUVmax and SULpeak changes categorized by clinical response. Clinical responders showed a significant larger ΔSUVmax(t_2_) and ΔSULpeak(t_2_) on ^18^F-FDG-PET (*P* = 0.030 and 0.003) than non-responders. The ΔSUVmax(t_1_) and ΔSULpeak(t_1_) of ^18^F-FDG-PET was not significantly different between clinical responders and non-responders. The ΔSUVmax(t_1_), ΔSULpeak(t_1_), ΔSUVmax(t_2_), and ΔSULpeak(t_2_) on ^18^F-FLT-PET were also not significantly different between these two groups.

**Fig. 2:**
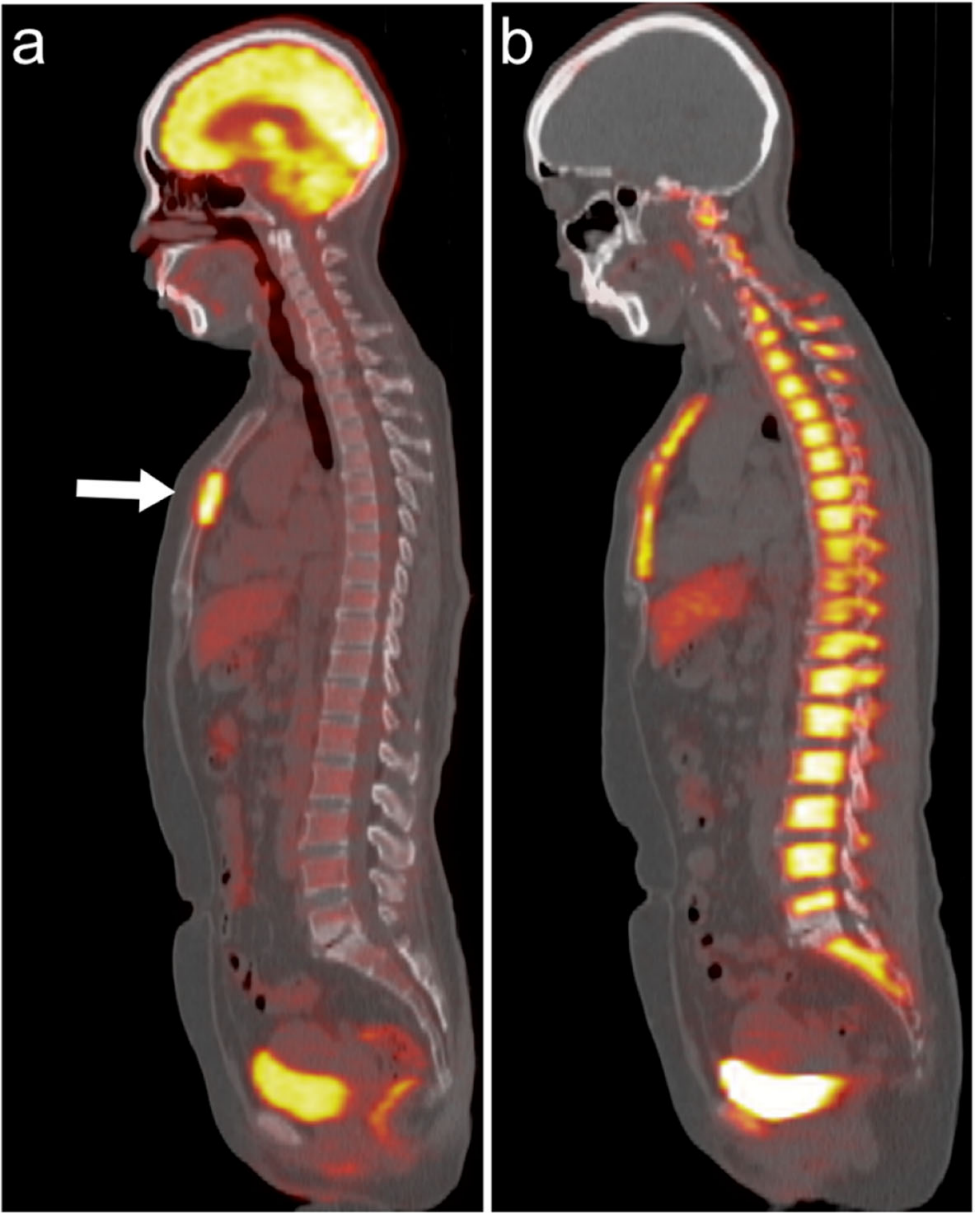
A case with metastatic breast cancer at the sternum. (**A**) ^18^F-FDG-PET, (**B**) ^18^F-FLT-PET. The lesion was clearly demarcated in ^18^F-FDG-PET. In ^18^F-FLT-PET, the margin of the lesion could not be well defined due to physiological ^18^F-FLT uptake in the adjacent bone marrow

The ratio of the SUVs and SULs of metastatic lesions to background uptake is demonstrated in Table 2. Figure 2 demonstrates a case with different target-to-background ratios in the bone marrow lesion on ^18^F-FLT-PET and ^18^F-FDG-PET.

**Table 2:** Target-to-background ratio of the SUVs and SULs of metastatic lesions in baseline ^18^F-FDG-PET and ^18^F-FLT-PET

Metastatic site	Units	Bone	Lung	Liver	Other
^18^F-FDG-PET	SUVs	5.94 ± 4.35	10.81 ± 15.32	3.77 ± 2.57	17.39 ± 21.05
	SULs	6.01 ± 4.40	9.22 ± 10.44	2.76 ± 1.85	11.91 ± 9.43
^18^F-FLT-PET	SUVs	1.90 ± 1.91	5.81 ± 4.09	1.00 ± 0.42	10.38 ± 8.23
	SULs	2.70 ± 2.67	4.96 ± 2.68	1.04 ± 0.27	7.40 ± 5.56

Post-1c or post-2c: after one or two cycles of chemotherapy, respectively.

### The predictive power of interim ^18^F-FLT-PET and ^18^F-FDG-PET for clinical response

**Fig. 3:**
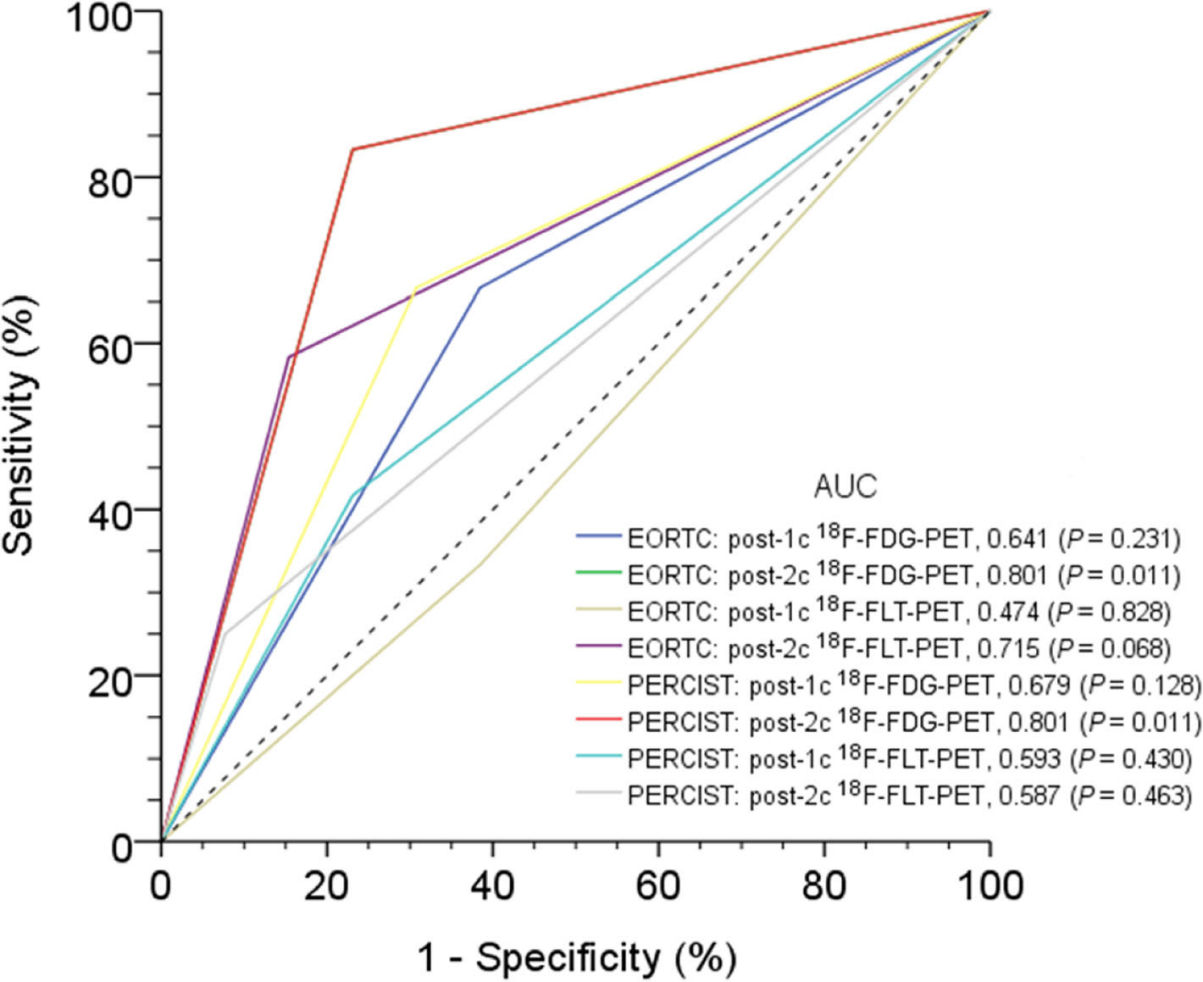
Areas under the receiver operating characteristic curves (AUCs) for post-1c or post-2c ^18^F-FDG-PET and ^18^F-FLT-PET for predicting clinical response based on EORTC criteria or PERCIST. The post-2c ^18^F-FDG-PET had a higher AUC value (*P* = 0.011)

**Fig. 4:**
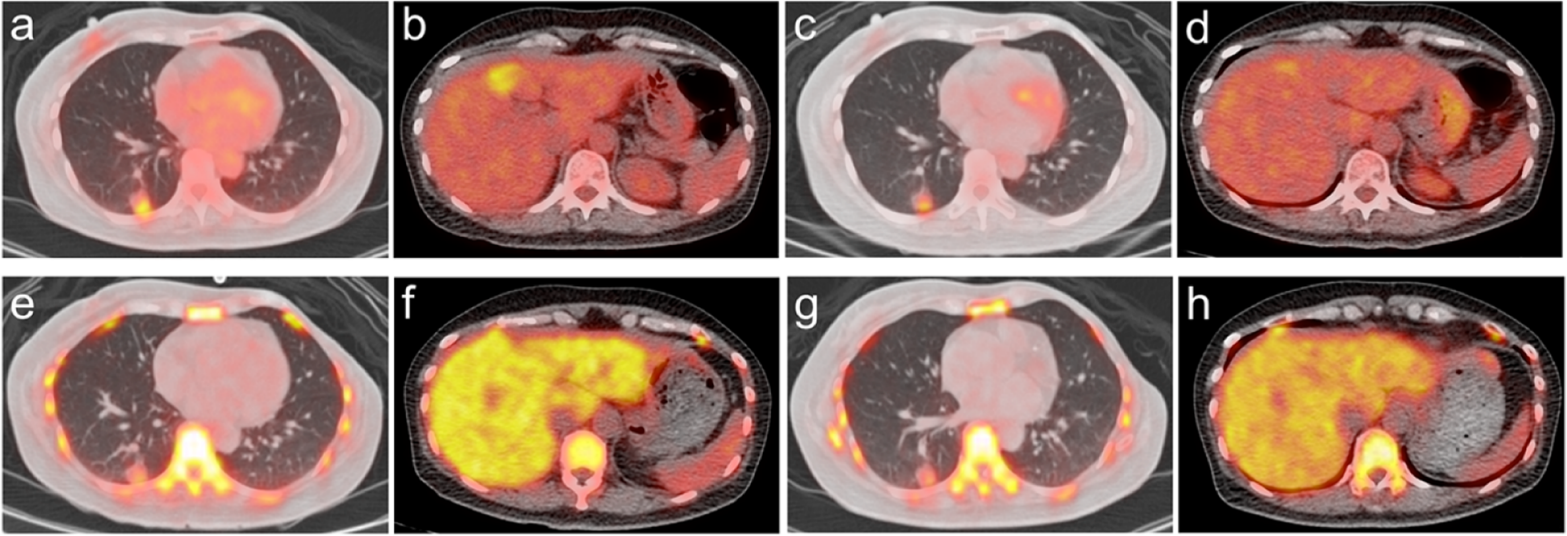
A case of a 51-year-old female presenting with metastatic breast cancer at the right lower lung and liver. (**A, B**) baseline ^18^F-FDG-PET, (**C, D**) post-2c ^18^F-FDG-PET, (**E, F**) baseline ^18^F-FLT-PET, and (**G, H**) post-2c ^18^F-FLT-PET

Figure 3 illustrates the ROC curves of ^18^F-FLT-PET and ^18^F-FDG-PET according to the EORTC or PERCIST criteria in predicting the clinical response. The AUC values of post-one cycle of chemotherapy (post-1c) and post-two cycles of chemotherapy (post-2c) ^18^F-FLT-PET were 0.474 and 0.715 based on the EORTC criteria, and 0.593 and 0.587 using the PERCIST criteria, respectively. The AUC values of post-1c and post-2c ^18^F-FDG-PET were 0.641 and 0.801 according to the EORTC criteria, and 0.679 and 0.801 using the PERCIST criteria, respectively. The predictive power of metabolic response in post-2c ^18^F-FDG-PET according to either PERCIST or EORTC criteria was statistically significant in predicting the clinical response (*P* = 0.011 for each criteria). The predictive capacities of ^18^F-FLT-PET did not reach significance. Figure 4 demonstrates a case with baseline and post-2c ^18^F-FDG-PET and ^18^F-FLT-PET images.

The hepatic lesion was poorly visualized on ^18^F-FLT-PET in the liver due to high physiological uptake. This patient received a docetaxel and cisplatin regimen. The post-2c ^18^F-FDG-PET revealed the SULpeak of the target tumor was decreased by 37.4%, which was compatible with partial metabolic response. The corresponding post-2c ^18^F-FLT-PET showed the SULpeak was decreased by 12.4%, indicating stable metabolic disease. She achieved partial response in the CT 3 months after the start of therapy. Post-2c ^18^F-FDG-PET was more accurate than ^18^F-FLT-PET in predicting the clinical response.

### ^18^F-FLT-PET versus ^18^F-FDG-PET metabolic response in predicting progression-free and overall survival

**Fig. 5:**
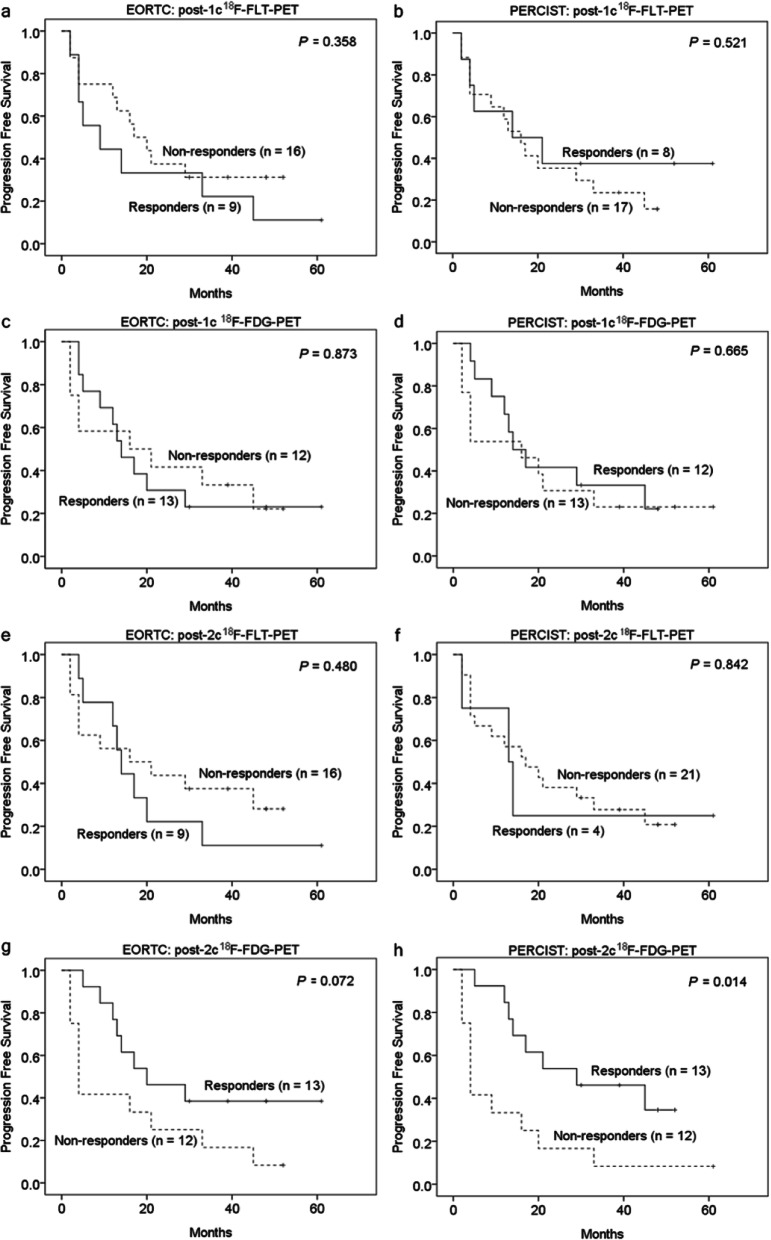
The Kaplan–Meier curves for the progression-free survival rate of metastatic breast cancer patients stratified by the response on ^18^F-FLT-PET or ^18^F-FDG-PET after one or two cycles of chemotherapy. The metabolic responders on post-2c ^18^F-FDG-PET based on PERCIST showed a significantly higher survival rate than metabolic non-responders

**Fig. 6:**
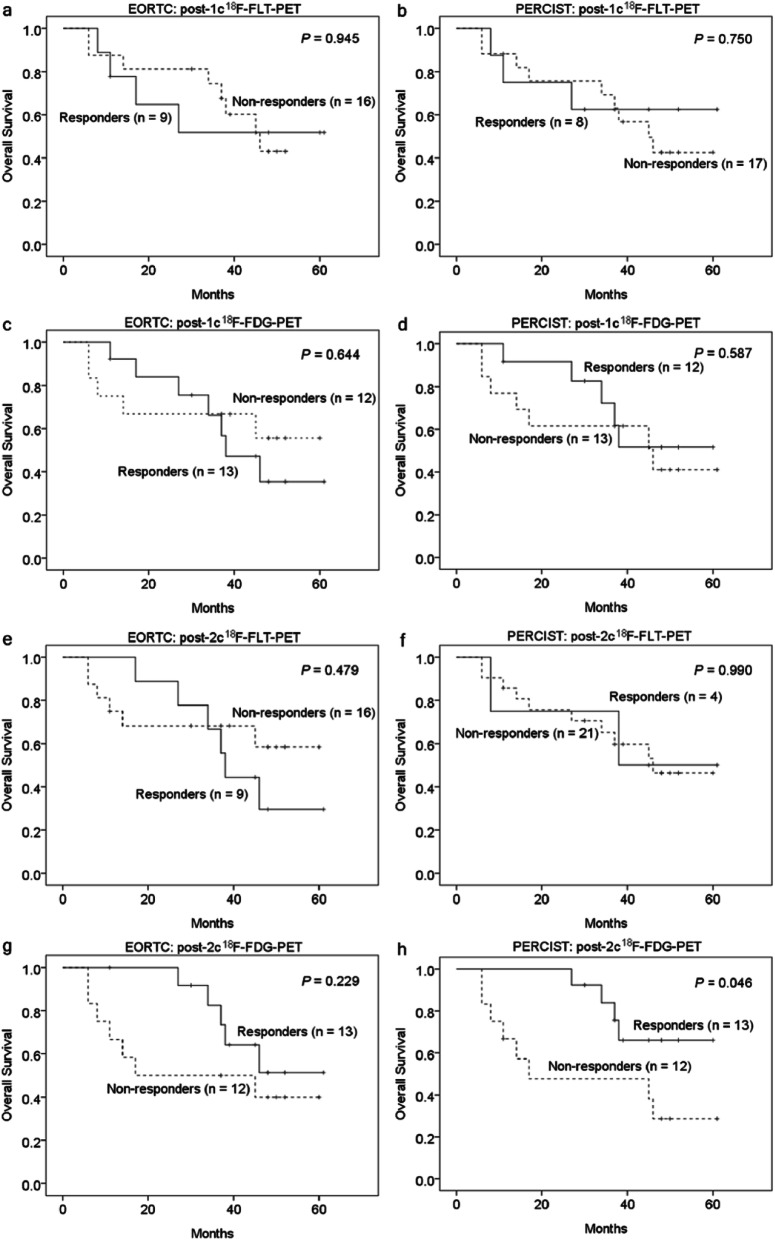
The Kaplan–Meier curves for the overall survival rate of metastatic breast cancer patients stratified by the response on ^18^F-FLT-PET or ^18^F-FDG-PET after one or two cycles of chemotherapy. The metabolic response on post-2c ^18^F-FDG-PET based on PERCIST significantly predicted the overall survival rate in these patients

Figures 5 and 6 demonstrate the comparison of PFS and OS between metabolic responders and non-responders using different PET criteria. The difference in PFS based on the post-1c or -2c ^18^F-FLT-PET response was not statistically significant. However, patients who were classified as post-2c ^18^F-FDG-PET responders using PERCIST had a significantly higher 2-year PFS than non-responder (53.8% vs. 16.7%; *P* = 0.014; HR = 0.335, 95% CI = 0.132–0.850). The post-1c ^18^F-FDG-PET response was not predictive of PFS.

We observed similar results in the analysis of metabolic response and OS. Patients who were classified as post-2c ^18^F-FDG-PET responders based on PERCIST had a significantly longer 2-year OS than non-responders (100% vs. 47.6%; *P* = 0.046; HR = 0.312**,** 95% CI = 0.093–1.048). The ^18^F-FLT-PET response was not able to predict OS.

Clinical responders determined by the RECIST criteria were not predicted to have a better OS (*P* = 0.091).

## Discussion

Because patients with mBC present with a low survival rate despite the development of new therapeutic regimens, it is critical to identify an imaging modality capable of early prediction of treatment outcomes, facilitating an individualized treatment. ^18^F-FDG-PET and ^18^F-FLT-PET have been advocated as promising tools in the early assessment of treatment outcome of cancer patients. However, there is still a lack of studies comparing the prognostic value of these two imaging modalities. In this study, we found that the ΔSUVmax and ΔSULpeak between clinical responders and non-responders were significantly different on post-2c ^18^F-FDG-PET but not on ^18^F-FLT-PET. The metabolic response on post-2c ^18^F-FDG-PET had a high predictive capacity (AUC = 0.801) for the clinical response. The PERCIST-defined metabolic responders on post-2c ^18^F-FDG-PET had a significantly longer PFS and OS than non-responders. In contrast, the metabolic response determined by interim ^18^F-FLT-PET failed to predict survivals. The interim ^18^F-FDG-PET demonstrated a higher prognostic value than ^18^F-FLT-PET in metastatic breast cancer.

Recently, research attempting to optimize cancer treatment has focused its attention on the potential utility of interim ^18^F-FDG-PET during therapy. ^18^F-FDG-PET has been shown to be beneficial in predicting the response to neoadjuvant therapy in locoregionally advanced breast cancer patients [22, 23]. Based on the literature, a greater decrease in SUV following the early cycles of neoadjuvant therapy is associated with a better histopathological status. As for mBC, Couturier et al. prospectively analyzed the role of interim ^18^F-FDG-PET for predicting treatment outcome and found that the metabolic response based on the EORTC criteria after the third cycle of chemotherapy significantly predicted both the clinical response and overall survival [24]. However, the PET response criteria used in previous studies were not consistent. Ridel et al. evaluated the association of the PET response determined by the PERCIST and the survival of mBC patients and reported that metabolic response was a superior predictor than response on CE-CT [25]. In our study, the data were analyzed using both PERCIST and EORTC criteria. Our findings further support the value of ^18^F-FDG-PET, showing that the metabolic response on post-2c ^18^F-FDG PET had a high AUC of 0.801 in predicting the clinical response. Moreover, the PERCIST-defined response of post-2c ^18^F-FDG-PET significantly prognosticated PFS and OS. Based on the results of previous studies and this study, interim ^18^F-FDG-PET is a promising tool for early prediction of the treatment response. mBC patients identified as metabolic responders had a better long-term prognosis, and the continuation of the treatment strategy in these patients seems reasonable. Conversely, in patients identified as non-responders, the therapy could be switched to other chemotherapy, target therapy, or immunotherapy and unnecessary toxicities due to futile treatment could be avoided.

FLT is phosphorylated by thymidine kinase-1, trapped within proliferating cells via the salvage pathway, but not incorporated during DNA synthesis [26]. FLT has been accepted as an imaging marker of cells in the S-phase of the cell cycle and is suggested to reflect tumour proliferation, aggressiveness, or response to therapy [27]. Because ^18^F-FLT is directly associated with tumour proliferation and does not substantially accumulate in inflammatory tissue [12], it has been considered a more reliable tracer than ^18^F-FDG in assessing the response after therapeutic intervention in cancer patients. The ability of ^18^F-FLT-PET as an early predictor for treatment response in breast cancer has been investigated in some previous studies [28–30]. Contrary to expectations, in our study with a pure mBC cohort, we found that the AUC values of interim ^18^F-FLT-PET for predicting the clinical response were not high. Moreover, the survival differences between metabolic responders and non-responders on the interim ^18^F-FLT-PET were not significant. The lack of prognostic significance of ^18^F-FLT-PET in our study may be attributed to the low target-to-background ratio in the liver and bone marrow (Table 2 and Fig. 2). The liver and bone are the predominant sites for distant metastases in breast cancer. The target-to-background ratio of ^18^F-FLT-PET in bone or liver lesions ranged from 0.2–6.8, which is substantially lower than the ratio in lesions at the lungs and other organ sites. The low target-to-background ratio in distant metastases may have interfered with the accuracy of lesion detection and ROI depiction. This could partially explain the discrepant results between our study and previous ones [10, 28–30], since these studies focused on patients with locally advanced breast cancer, which has a high target-to-background ratio. Another reason for the discrepant results may be the use of different therapeutic agents across studies [31].

Head-to-head comparison of ^18^F-FDG-PET with ^18^F-FLT-PET in the early evaluation of treatment response has been addressed in other malignancies but shows inconsistent results. Crandall et al. compared the predictive power of ^18^F-FLT-PET with that of ^18^F-FDG-PET in patients with non-small cell lung cancers receiving neoadjuvant chemotherapy [15]. They found that a significant decrease of ^18^F-FDG uptake after one cycle of chemotherapy and ^18^F-FDG-PET had a high AUC of 0.91 in predicting anatomic tumour response. In contrast, the decrease of ^18^F-FLT did not differ significantly between responders and non-responders. Rendl et al. investigated the prognostic value of SUV changes on ^18^F-FLT-PET and ^18^F-FDG-PET performed at baseline and post neoadjuvant chemotherapy in rectal cancer [13]. In that study, the SUV changes of both imaging modalities could not reliably separate histopathological responders from incomplete responders. Differences in the timing of interim PET and the type of cancer may explain the inconsistent results among these reports. More research on this topic needs to be undertaken before the comparative prognostic values of ^18^F-FLT-PET and ^18^F-FDG-PET can be clearly understood.

The present study has some limitations. First, the study population was relatively small, which limits the strength of our results. Therefore, larger independent trials are required to validate our findings. Second, not all concerned lesions were pathologically confirmed. However, it is a common issue in this setting and increasing it is often neither feasible nor justified ethically. Finally, some of the patients with positive hormone receptor status had not received endocrine therapy before enrollment in this study due to potential visceral crisis condition or history of hormone therapy failure. Hence, the result might have been confounded by the different treatment regimens prescribed. However, the regimen prescribed in our study was adhered to international guideline and real-world practice, which highlights the potential clinical value of this study. These limitations notwithstanding, our data may have important implications for mBC patients. Specifically, our findings may represent a valuable addition to the current literature in light of the increasing use of interim PET.

## Conclusions

In this head-to-head comparison study, the metabolic response on interim ^18^F-FDG-PET showed a high predictive capacity for clinical response and survival outcome in patients with mBC. Interim ^18^F-FLT-PET demonstrated an inferior prognostic value. The low target-to-background ratio of ^18^F-FLT in metastatic lesions in the liver or bone may explain the discrepant results. ^18^F-FLT-PET should be used with caution when the target tumour is located in these organs. Interim ^18^F-FDG-PET is more suitable than ^18^F-FLT-PET in selecting mBC patients who will benefit from systemic chemotherapy or identifying those at risk of treatment failures early, permitting treatment individualization and consideration of alternative strategies.

## Data Availability

The data sets analyzed during this study are available from the corresponding author on reasonable request depending on IRB approval.

## Abbreviations

AUC: Area under the ROC curve
CE-CT: Contrast-enhanced CT
CI: Confidence intervals
CMR: Complete metabolic response
CR: Complete response
CT: Computed tomography
EORTC: European Organization for Research and Treatment of Cancer
ER: Estrogen receptor
^18^F-FDG: ^18^F-fluorodeoxyglucose
^18^F-FLT: ^18^F-fluorothymidine
HER-2: Human epidermal growth factor receptor 2
mBC: Metastatic breast cancer
OS: Overall survival
PD: Progressive disease
PET: Positron emission tomography
PERSIST: PET Response Criteria in Solid Tumors
PFS: Progression-free survival
PMD: Progressive metabolic disease
PMR: Partial metabolic response
PR: Partial response
PR: Progesterone receptor
RECIST: Response Evaluation Criteria in Solid Tumors
SD: Stable disease
SMD: Stable metabolic disease
SULpeak: Peak SUV normalized by lean body mass
SUV: Standardized uptake value
VOI: Volume of interest
